# Characterization of the Stages of Creative Writing With Mobile EEG Using Generalized Partial Directed Coherence

**DOI:** 10.3389/fnhum.2020.577651

**Published:** 2020-12-07

**Authors:** Jesus G. Cruz-Garza, Akshay Sujatha Ravindran, Anastasiya E. Kopteva, Cristina Rivera Garza, Jose L. Contreras-Vidal

**Affiliations:** ^1^Laboratory for Non-Invasive Brain-Machine Interface Systems, NSF IUCRC BRAIN, University of Houston, Houston, TX, United States; ^2^Department of Hispanic Studies, University of Houston, Houston, TX, United States

**Keywords:** creative writing, creativity, EEG, MoBI, generalized partial directed coherence

## Abstract

Two stages of the creative writing process were characterized through mobile scalp electroencephalography (EEG) in a 16-week creative writing workshop. Portable dry EEG systems (four channels: TP09, AF07, AF08, TP10) with synchronized head acceleration, video recordings, and journal entries, recorded mobile brain-body activity of Spanish heritage students. Each student's brain-body activity was recorded as they experienced spaces in Houston, Texas (“Preparation” stage), and while they worked on their creative texts (“Generation” stage). We used Generalized Partial Directed Coherence (gPDC) to compare the functional connectivity among both stages. There was a trend of higher gPDC in the Preparation stage from right temporo-parietal (TP10) to left anterior-frontal (AF07) brain scalp areas within 1–50 Hz, not reaching statistical significance. The opposite directionality was found for the Generation stage, with statistical significant differences (*p* < 0.05) restricted to the delta band (1–4 Hz). There was statistically higher gPDC observed for the inter-hemispheric connections AF07–AF08 in the delta and theta bands (1–8 Hz), and AF08 to TP09 in the alpha and beta (8–30 Hz) bands. The left anterior-frontal (AF07) recordings showed higher power localized to the gamma band (32–50 Hz) for the Generation stage. An ancillary analysis of Sample Entropy did not show significant difference. The information transfer from anterior-frontal to temporal-parietal areas of the scalp may reflect multisensory interpretation during the Preparation stage, while brain signals originating at temporal-parietal toward frontal locations during the Generation stage may reflect the final decision making process to translate the multisensory experience into a creative text.

## 1. Introduction

Creative writing involves embodied practices that physically and emotionally connect us with our surroundings (Rivera Garza, 2013). We investigated creative writing as a bodily experience, in which the author's interaction with the world around them (physically, verbally, perceptually, and emotionally) informs the preparation and elaboration of their written work. In this way, as authors actively seek and engage in experiences in the world around them through their body and mind, these experiences affect the aesthetic and semantic components of their creative output.

Previous studies have used scalp electroencephalography (EEG) to analyze neural correlates of writing. For example, in a study using a custom test to evaluate reading and writing achievement to assess educational grade requirements, Harmony et al. (1990) found that high correlation in the delta and theta bands in frontal and temporal electrodes was related to poor writing performance, while high correlation in the alpha band in occipital areas is related to high writing performance in a group of 81 children. The same research group (Marosi et al., 1995) reported high coherence in delta (1–4 Hz), theta (4–8 Hz), and beta (12–24 Hz) bands associated with poor performance in reading and writing in 84 children. Conversely, high coherence in the alpha (8–12 Hz) band was related to proficient reading and writing. Coherence metrics between pairs of EEG electrodes in different scalp areas have been shown to have a positive correlation with an individual's creativity level, in short creative verbal and visual tasks (Petche et al., 1997), and in the Torrance Test of creative thinking (Jaušovec and Jaušovec, 2000a). These findings suggest that coherence has a more intense relationship with creative performance than EEG frequency-band-power metrics.

Neuroimaging studies with functional magnetic resonance imaging (fMRI) have been used to measure functional connectivity (FC) of participants at rest, the resting state FC (rFC). Lotze et al. (2014) found decreased rFC between inter-hemispheric areas BA 44, and left area BA 44 with the left temporal lobe for individuals who scored higher in a verbal creativity index test. These brain areas are part of Broca's area, a region located in the frontal part of the left hemisphere of the brain that is active in semantic tasks, such as semantic decision tasks (determination of whether a word represents an abstract or a concrete entity) and generation tasks (generation of a verb associated with a noun).

In studies where participants generated original text compositions, fMRI studies have also analyzed the human creative process through its distinct stages of preparation, generation, and revision. Shah et al. (2013) studied the Preparation and Generation stages, by the implementation of an actual creative writing paradigm. They found distinct cortical networks associated with each stage in a fronto-parieto-temporal network. The Preparation stage, “brainstorming,” was found to activate the premotor cortex (involved in the cognitive-motor preparation to write), language processing areas in the bilateral IFG and left temporal areas, and left lateral orbito-frontal regions for higher order cognitive processing. Creative writing activated areas associated with handwriting (primary motor cortex and somatosensory areas), and cognitive processing such as episodic memory retrieval and semantic integration in bilateral hippocampi and temporal poles, with right-lateralized activation in posterior and anterior lobes. The same research group found that expert writers had higher activation in medial prefrontal cortex (mPFC) and basal ganglia areas (Erhard et al., 2014). Liu et al. (2015) studied the generation and revision stages and reported that the mPFC was active during both stages and the responses in dorsolateral prefrontal cortex (DLPFC) and Intraparietal sulcus (IPS) were deactivated during the Generation stage.

Differences in brain activity for the distinct stages of the creative process however, remain mostly unexplored in the EEG domain; particularly for creative writing tasks. As fMRI studies represent indirect evidence about cortical dynamics in cognitive-motor tasks such as creative writing, it is important to use time-resolved, direct methods that assess brain dynamics in action and in context in natural settings. In this regard, mobile EEG allows for the collection of brain activity data in more natural settings, where the users have freedom of motion and can freely walk around their surroundings (Cruz-Garza et al., 2019).

To better understand the brain dynamics of writers working 'in action and in context' in both preparatory and generative stages of creative writing, we integrated wearable MoBI technology into a creative writing course in Spanish at the University of Houston. The course was designed and led by Prof. Cristina Rivera Garza at the University of Houston. The experiment was designed together with the aims of the course to provide an equal consideration in the experimental design and evaluation process to best assess the creative process in an authentic creative writing experience.

Specifically, we studied the process of creative writing with eighteen Spanish heritage speakers, as they engaged in the Preparation and Generation stages of their writing. The students were asked to walk through different areas of the City of Houston to experience a variety of pre-selected contextual environmental settings (we called them “prompts”) chosen by the instructor, and use the experience to provide aesthetic and semantic content in their narratives. This study aimed to use scalp electroencephalography (EEG) to identify brain features or neural markers related to the different stages of creative writing where free behaving participants were able to move, explore their surroundings to inform their creative texts (Preparation stage), and write at their own time (Generation stage).

This study investigates the neural features associated with creative writing using quantitative and mobile EEG, through the characterization of the Preparation and Generation of creative texts in students that participated in a creative writing workshop. The students developed their writing skills throughout the workshop, and physically interacted with space and their communities during the Preparation stage. The Generation stage consisted of creating a first draft for each of their assignments.

## 2. Methods

### 2.1. Human Participants

Eighteen heritage Spanish speaking undergraduate students participated in a Spanish language creative writing upper-division undergraduate workshop (SPAN 3308 YOUR BRAIN ON WRITING: Writing, Body, and Neuroaesthetics) at the University of Houston. The participants provided Anonymous Informed Consent, approved by the University of Houston Institutional Review Board, at the beginning of the workshop. The students received training to set up their own EEG headsets and body-mounted video cameras for the experiment. The students were responsible for the collection of EEG data, video, and to keep a diary with notes on each recording session.

### 2.2. Experimental Task

Through readings and writing prompts, participants were asked to experience and acknowledge the physicality of the writing process and to relate it to the materiality of language. Prompts, designed by the instructor, encouraged students to develop and record a series of specific writing preparation tasks that emphasized the physicality of the participants' bodily experience (e.g., walking, running, climbing in different locations of Houston) as part of the required assignments for the workshop. The writing prompts are provided in the Supplementary Materials.

The participants were then asked to utilize these prompted experiences to generate their creative texts in a 3–5 page suggested draft length (double space, 11 point font). The students were instructed to use the EEG and video cameras during both their prompted physical activities and writing time. There could be more than one session of walking and writing EEG recording sessions per prompt.

**Figure 1:**
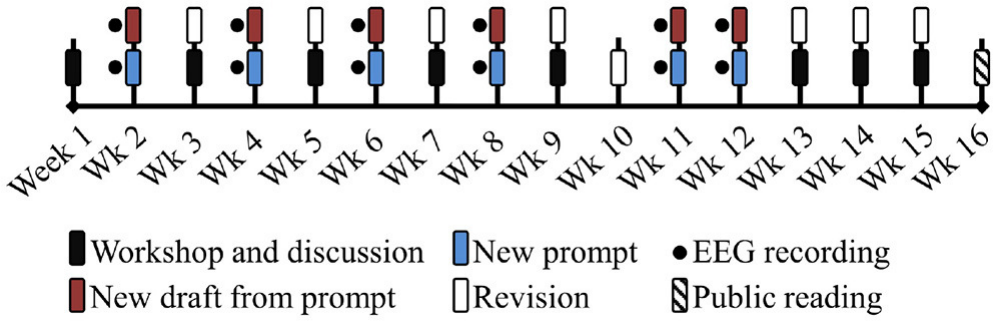
Creative writing workshop 1. Pilot study. Timeline for the EEG recording sessions in the creative writing workshop: *Talleres de escrituras*.

A timeline schematic of the creative writing workshop is represented in Figure 1. The workshop was 16 weeks long, with six creative writing exercises distributed in weeks 2, 4, 6, 8, 11, and 12. In the weeks in between the creative writing sessions, the students would discuss their peers' texts and improve their own previous drafts. At the end of the workshop, there was a public reading of the finalized creative texts.

This experimental setup produced data in two stages of the creative process: the Preparation stage and the Generation stage. The Preparation stage involved tasks such as walking, active observation of their environment, taking notes, and ideation. For the Generation stage, the task involved reviewing their notes and creating a creative narrative, with iterative revisions and modifications.

### 2.3. Equipment

EEG and head acceleration data were captured using Muse headsets (Interaxon, Toronto, Ontario, Canada). The headset has seven sensors, two out of these seven sensors are positioned at the frontal region (AF07 and AF08), two at temporal-parietal region (TP09 and TP10), and the remaining three sensors served as electrical reference located at the center of the forehead (Fpz). The 61 g headset has a built-in accelerometer that was used to measure the head acceleration. EEG data for each channel are measured in microvolts (μV) with a sampling rate of 220 Hz at 10-bit resolution. The acceleration data was recorded at 50 Hz. Additionally, the data recordings contain a vector indicating contact quality (sampled at 10 Hz) for each electrode, rating contact quality as “indicator = 1: good,” “indicator = 2: acceptable,” “indicator ≥ 3: bad.” (see http://developer.choosemuse.com/hardware-firmware/hardware-specifications for full technical specifications). Previous experiments have shown the capacity of the Muse headsets for the collection of mobile EEG data outside a laboratory setting (Ravindran et al., 2019).

**Figure 2:**
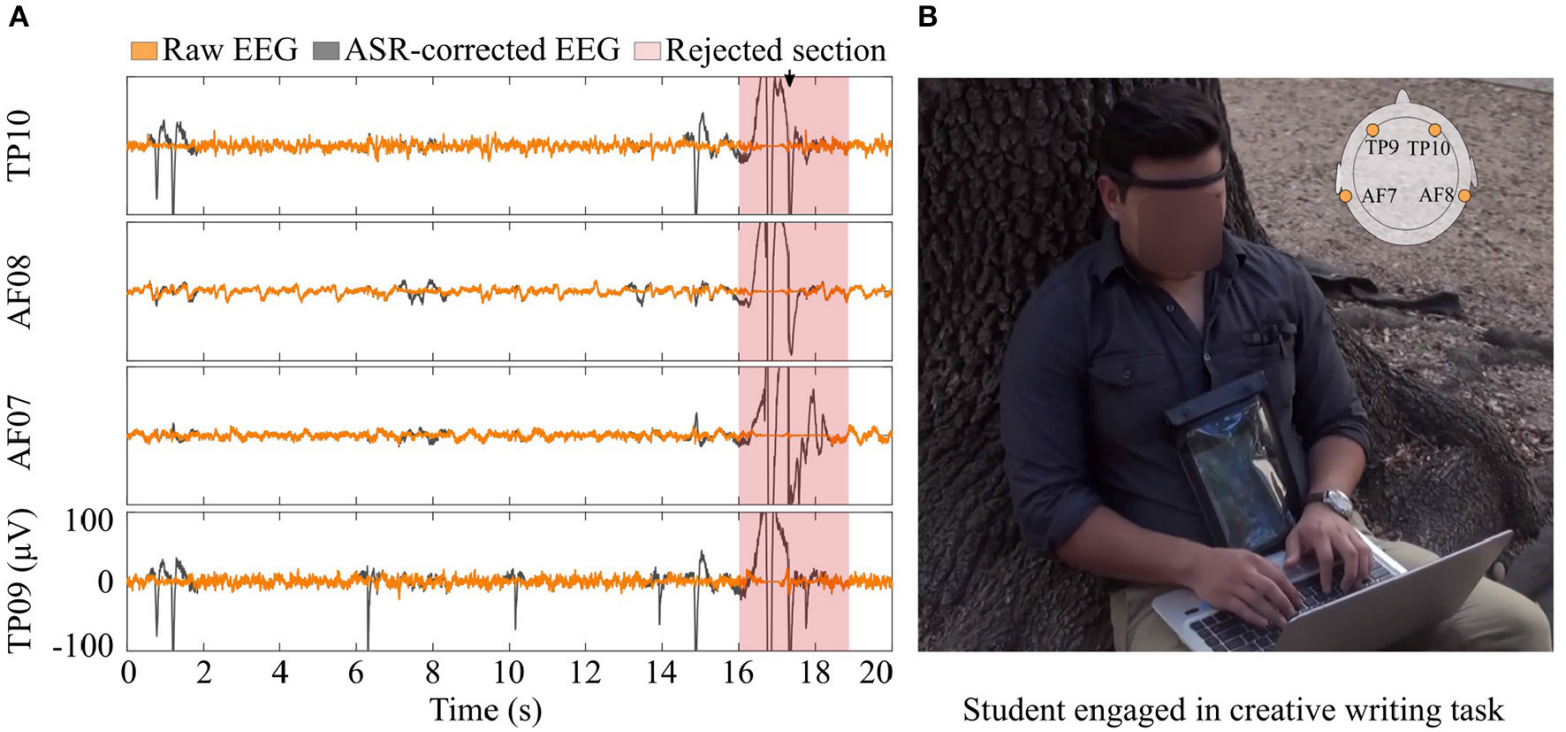
Equipment setup and EEG data pre-processing. **(A)** Raw (black) and pre-processed (orange) EEG data. Shaded areas indicate rejected intervals. **(B)** A student wearing the EEG headset during the Generation stage.

The participants set up their own headset with a custom application given to them in a personal tablet, which recorded EEG and head acceleration data and labeled the participant identification number and date/time for the recording session automatically. The data recording setup is illustrated in Figure 2. Additionally, the participants set up body-cameras (Conbrov, ShenZhen, China) to record their exploration (Preparation) and writing (Generation) sessions. The camera recorded 720 HD video on a 75° wide-angle ens.

### 2.4. Data Collection

The students were asked to make five writing exercises and collect their brain activity as they walked and observed their environment (Preparation stage), and created their texts (Generation stage). Only writing assignments that were submitted and accompanied by both video and EEG data were considered for the analysis. From the 18 participants, data from eleven students was discarded due to incomplete data (video or EEG missing) or assignments not submitted on time. This represents a yield of 39% of the total data collected for further analysis.

The Preparation and Generation stages for each writing exercise were done in several distinct recording sessions as each stage could take several recording sessions to complete during the semester. We kept each data recording as a separate session to analyze. Recording sessions were considered for analysis when all four electrodes had a “good” contact indicator for at least one continuous minute of data.

### 2.5. Pre-processing

Data recordings with both video (providing contextual cues) and EEG were considered for this analysis. An online notch filter was applied on the EEG data to remove the 60 Hz power line noise (available as a user preset for the headband). We applied an offline 4th order, zero-stage Butterworth band-pass filter from 1 to 100 Hz. Artifact Subspace Reconstruction (ASR) (Mullen et al., 2013) was used for the removal of short-time high-amplitude artifacts in the continuous data as in Ravindran et al. (2019). Calibration data for ASR for each student was computed from the entire length of the trial using automated methods. A cut off threshold of ten standard deviations was used for the identification of corrupted subspaces, and a window length of 500 ms with a step size of 250 ms was used for the ASR. Among the segments, channels having corrupt PC loading to be >0.75 were removed. The remaining segments were then inspected automatically to remove data from any electrode disconnections from the scalp (tracked by the headband status data), any abrupt change of voltage >100 μV, or EEG data collected while there was an absolute acceleration magnitude larger than 1 ms^−2^ (Ravindran et al., 2019). After removing noisy segments of data with our pre-processing methods, 77% of the data was kept for analysis.

### 2.6. Feature Extraction

#### 2.6.1. Functional Connectivity, FC

Functional Connectivity is defined as the statistical association among two or more anatomically distinct time-series and can be assessed with EEG coherence measures or fMRI (Friston et al., 1993). FC analysis was performed upon the EEG channels by computing the generalized partial directed coherence (gPDC) measure (Baccala et al., 2007) over 6 s time segments with 50% overlap, a short-time based stationary approach (Omidvarnia et al., 2011). Partial coherence measures have been found to perform well with low-density EEG (Barzegaran and Knyazeva, 2017), making it a useful tool for our EEG dataset of four channels. PDC is a frequency-domain metric that provides information about directionality in the interaction between two signals among a larger number of signals (Baccalá and Sameshima, 2001), but it is dependent on the scale of the individual inputs (Blinowska, 2011). To account for this, Baccala et al. (2007) introduced a normalization term based on the variance of the signals and denoted this modified measure as gPDC, which was used in this study with the FieldTrip implementation [ft_connecitivityanalysis()] (Oostenveld, 2020); with the BSMART toolbox (Cui et al., 2008). We chose a 6 s epoch due to observed stability at epoch lengths of 6 s or more for FC measures (Fraschini et al., 2016). gPDC was estimated using a multivariate autoregressive model (MVAR) using all four electrodes. We used an MVAR model order of 12 (54 ms), which was obtained by using the ARFIT algorithm (Schneider and Neumaier, 2001) and evaluating the SBC criterion, which is least affected by noise (Porcaro et al., 2009). The observed gPDC estimates were plotted for all pairs of electrodes in the frequency bands: delta [1–4 Hz], theta [4–8 Hz], alpha [8–12 Hz], beta [12–30 Hz], gamma [30–50 Hz].

#### 2.6.2. Power Spectral Density, PSD

The power spectral density (PSD) was computed for each data window using Thomson's multitaper PSD estimate, with 4,096 frequency bins [1–50 Hz] and half-bandwidth product nw = 4. The mean PSD was obtained for each recorded session. Those recorded sessions corresponding to the Preparation and the Generation stage were compared for each of the four electrodes separately.

#### 2.6.3. Sample Entropy, SampEn

Complexity measures has been used in different studies to measure levels of creativity before (Jaušovec and Jaušovec, 2000b; Shourie et al., 2014). Approximate Entropy is a measure of signal regularity which that explores the time ordering of data points by calculating the log likelihood that runs of pattern which are close remain close for incremental comparison (Pincus, 1995). Lower value of Approximate Entropy indicates that the signal is more regular or predictable. However, many studies have reported reliability issues using Approximate Entropy due to the self-match involved in Approximate Entropy computation leading to a bias (Richman and Moorman, 2000; Chen et al., 2006). A new metric called Sample Entropy (SampEn) (Richman and Moorman, 2000) was proposed aimed at reducing the bias of Approximate Entropy. The parameters remained the same for both Approximate Entropy and SampEn: the “filter factor” *r*, length of sequences being compared *m* and the signal length *N*. SampEn has shown to be relatively less dependent on the signal length and shows better stability for wider range of parameters (Richman and Moorman, 2000; Chen et al., 2006; Boskovic et al., 2012). Earlier studies showed that SampEn gives better statistical validity for *m* = 2 and the *r* in the range of 0.1–0.25 standard deviations (Richman and Moorman, 2000; Bruce et al., 2009; Zarjam et al., 2012). In this study, we used *m* = 2 and *r* = 0.2 standard deviations of the signal window, and *N* = 1, 320 (6 s of data sampled at 220 Hz). As with the PSD analysis, the SampEn means were computed for each recorded data collection session, and those corresponding to the Preparation and Generation Stages were compared for the four electrodes separately.

#### 2.6.4. Statistical Analyses

Statistical significance comparing the Preparation and Generation Stages was obtained using a two-tailed unpaired *T*-test with a significance level of *p* < 0.05. All comparisons show the mean, confidence intervals at the *p* < 0.05 significance level, and an indication for when the difference was statistically significant at that statistical level. The Fisher-Snedecor *F*-test was performed to assess if the variances were equal. When the variances were different (Cardillo, 2020), the Satterthwaite approximate *T*-test was performed (Satterthwaite, 1946).

## 3. Results

**Figure 3:**
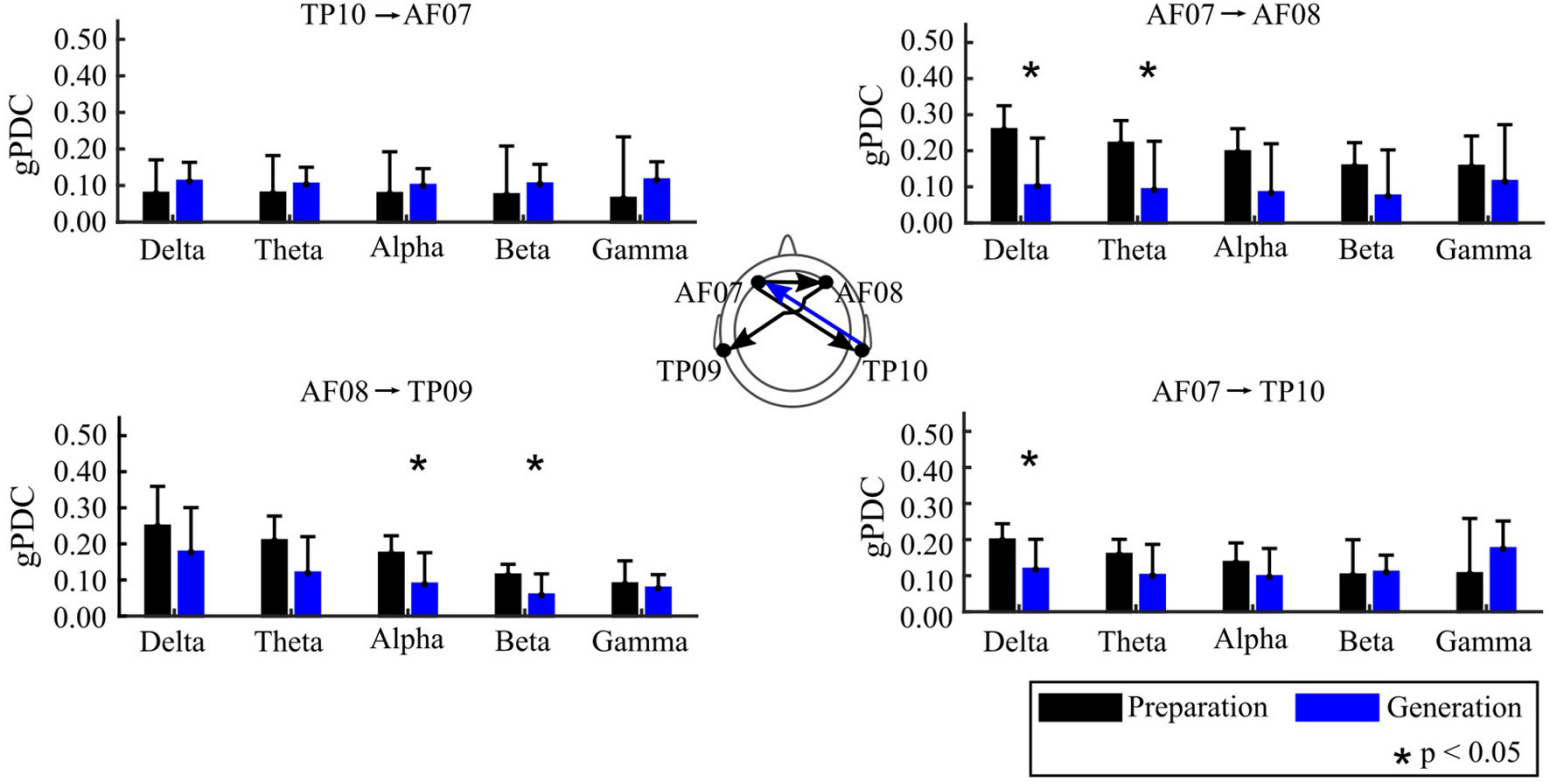
gPDC for the Preparation and Generation Stages, displaying the mean and 95% confidence intervals. The scalp map at the center displays the connections highlighted in the bar graphs; where the color of the arrow corresponds to the condition (creative writing Preparation or Generation) where gPDC was higher.

The main finding of this study is that the Preparation and Generation stages of creative writing were characterized differentially in terms of the functional connectivity among the scalp locations examined. Specifically, the gPDC between the Preparation and Generation stages showed the opposite directionality between right temporal and left anterior frontal areas. Figure 3 shows significant differences in FC between electrode pairs, using gPDC, during the two stages of the creative writing process analyzed.

Preparation stage: There was higher gPDC in the Preparation stage originating from anterior-frontal electrodes toward temporal-parietal electrodes. The gPDC difference between the Preparation and the Generation stages showed statistically broadband significance, at a confidence level of *p* < 0.05, for three connections. AF07 to TP10 showed significant differences in the delta (1–4 Hz) frequency range. There was statistically higher gPDC observed for the connections AF07 to AF08 in the delta and theta (1–8 Hz) frequency bands, and AF08 to TP09 from alpha (8–12 Hz) to beta (12–30 Hz). Figure 3 shows the gPDC values, bounded between 0 and 1, for those connections where statistical differences were found between conditions.

Generation stage: There was higher Partial Directed Coherence in the Generation stage originating from TP10 toward AF07. Although there was no significant differences in the TP10 to AF07 comparison (Figure 3) it was the only connection that showed higher gPDC for the Generation stage in a clear trend across frequency bands.

The statistical difference in gPDC and its opposite directionality when comparing the Preparation and the Generation Stages indicates that there was a strong functional relation between the left anterior frontal with the right temporal-parietal areas when the students engaged in the tasks.

**Figure 4:**
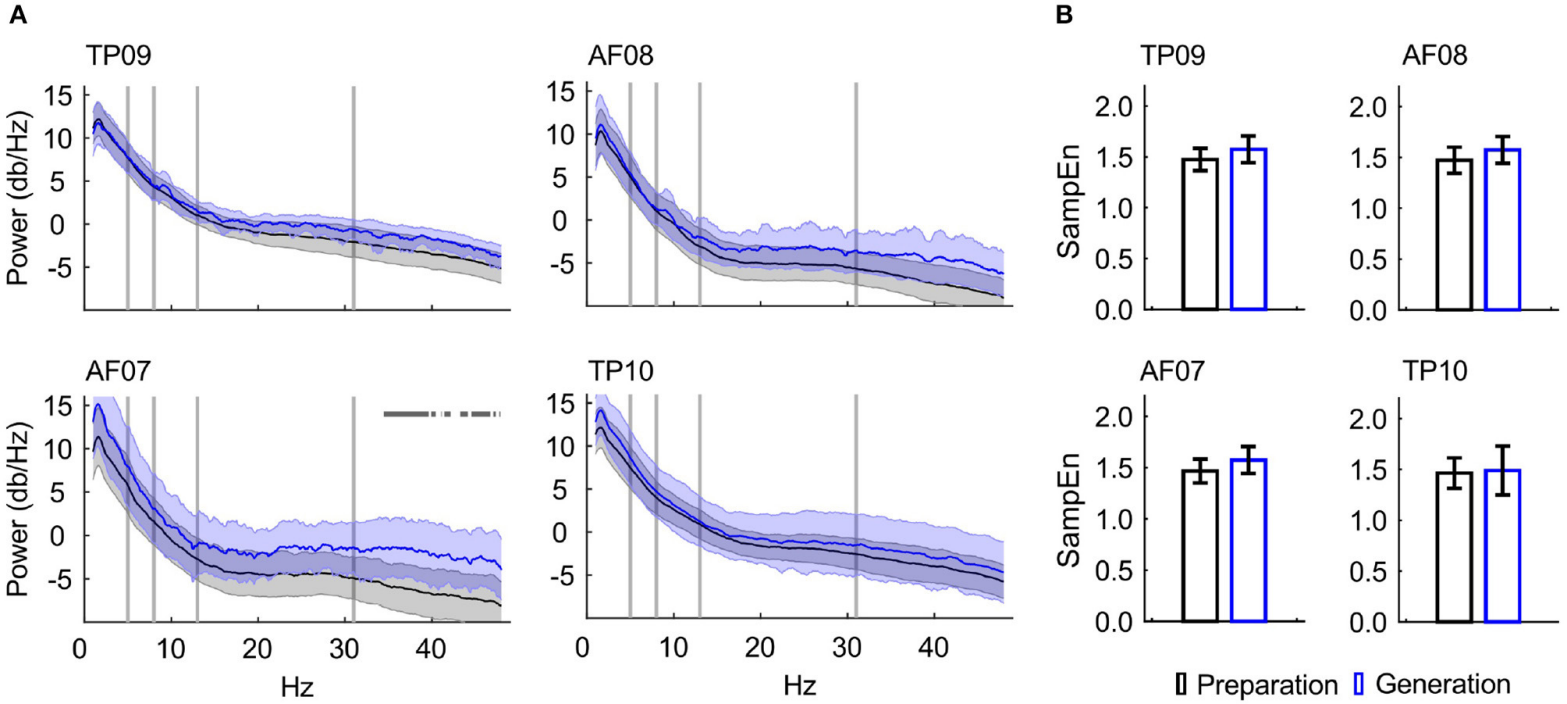
EEG feature characterization. **(A)** Power spectrum density for each EEG channel, displaying the mean and 95% confidence intervals. Vertical bars indicate selected frequency-band limits. Horizontal marks indicate statistical significant differences: AF07, 32–40 Hz. The numbers following the “Preparation” and “Generation” labels indicate the number of unique students' data contributed to the PSDs. **(B)** SampEn for the Preparation and Generation Stages, displaying the mean and 95% confidence intervals. No significant differences were found (*p* < 0.05). Electrode TP09 contained data from 30 EEG recordings, AF08 from 21 recordings, AF08 from 22 recordings, and TP10 from 18 recordings.

Frequency band-power analysis showed a statistically significant difference across writing stages within the gamma (32–40 Hz) frequency range for the AF07 electrode only (Figure 4A).

The SampEn was higher during Generation stage compared to Preparation stage, although this difference did not reach statistical significance (Figure 4B).

## 4. Discussion

The higher functional connectivity from anterior frontal scalp areas toward temporal-parietal areas during the Preparation stage suggests a functional relationship between areas involved in the processing of multisensory inputs (Perrodin et al., 2014; Schapiro et al., 2014) and episodic emotional memory integration (Dolcos et al., 2004, 2005; Lech and Suchan, 2013) in the temporal lobe as participants explore their surroundings actively engaging the frontal cortex in integrating the experience. The opposite directionality between the same electrodes (Figure 3) at the Generation stage reinforces this hypothesis in which sensory input are reprocessed in the frontal areas to produce a draft of a creative text based on those experiences.

Our results, although constrained to frontal and temporal recording locations, relate to previous findings in EEG and fMRI studies analyzing the different stages of the creative process. It furthers the suggestions from Petche et al. (1997) and Jaušovec and Jaušovec (2000a) that EEG correlates of creative performance are more pronounced in functional connections between brain areas than localized frequency-band power. In the last decade, distinct cortical networks have been associated with each stage of the human creative writing process. Shah et al. (2013) identified ventrolateral prefrontal cortex activation during the Preparation stage, and central-parietal areas involved in the Generation stage. “Brainstorming” engaged cognitive, linguistic, and creative functions represented in a parieto-frontal-temporal network, while “Creative writing” activated motor, visual, a cognitive and linguistic areas mainly over central and parietal networks (Shah et al., 2013). Liu et al. (2015) found that the mPFC was active during the generation and revision stages. They observed deactivation of the dorsolateral prefrontal cortex (DLPFC) and inferior parietal sulcus (IPS) during the Generation stage.

Our results show a trend of higher gPDC values from the right temporal toward the left anterior frontal electrode during the Generation stage of creative writing, for all frequency bands analyzed (1–50 Hz), albeit without reaching statistical significance; and the opposite directionality for the Preparation stage with statistical significance at 1–4 Hz. The Preparation stage also showed higher connectivity compared to the Generation stage with connections originating from anterior-frontal electrodes: AF07 to AF08 (significance at 1–8 Hz), and AF08 to TP09 (significance at 8–30 Hz). We did not find statistical differences between the Preparation and the Generation Stages for Sample Entropy; and frequency band-power showed differences only in the left anterior frontal electrode in the gamma band. In future studies, Entropy analysis could benefit from multi-scale (Gao et al., 2015) and multi-variate (Ahmed and Mandic, 2011) entropy models to account for the varying and complex nature of physiological signals (Costa et al., 2002).

Overall, these findings suggest that ideation, exploration, and observation during the Preparation stage of a creative writing task can be characterized by a state of long-range cortico-cortical communication between multisensory integration brain areas (temporal regions) and high-order execution and planning areas of the brain (prefrontal regions), perhaps leading to selective storage of ideas, concepts or observations candidate for creating writing during the Generation stage. We hypothesize this focal activity may be related to working memory, sequence production, and processing of filtered information from the Preparation stage.

## Data Availability Statement

The raw data supporting the conclusions of this article will be made available by the authors, without undue reservation.

## Ethics Statement

The studies involving human participants were reviewed and approved by University of Houston's Institutional Review Board (IRB). The participants provided their written informed consent to participate in this study.

## Author Contributions

JC-G performed the data analysis and wrote the manuscript. AS prepared and pre-processed the data, preformed preliminary analysis, and contributed with data-processing code. AK assisted students on a weekly basis on data collection, and compiled the multimodal data in a working dataset. JC-G, AK, and CR planned the experiment. CR conducted the workshop. JC-V and CR conceived the research and edited the manuscript. All authors reviewed the manuscript.

## Conflict of Interest

The authors declare that the research was conducted in the absence of any commercial or financial relationships that could be construed as a potential conflict of interest.

## Supplementary Material

The gPDC distributions for the data from all participants and creative writing exercises used to generate Figure 3 can be found as Supplementary Materials.
