# Supplementary material for: Characterization of the Stages of Creative Writing With Mobile EEG Using Generalized Partial Directed Coherence
Source: Front Hum Neurosci. 2020 Dec 7;14:577651. doi: 10.3389/fnhum.2020.577651 (PMC7793781; doi:10.3389/fnhum.2020.577651)
Supplement: Supplementary file 1 [file Data_Sheet_1.PDF]

Supplementary Material

1 CREATIVE WRITING PROMPTS

EE#1: A summer weekend walk in the afternoon, Houston

Remember Solnit's phrase while describing the walk of Virginia Wolf during the London winder: she goes out in the afternoon (4 to 6pm) to buy a pencil, but in reality she does it to explore the darkness. She walks aimlessly, she identifies objects, she describes them carefully. To do something similar in Houston at the end of the summer, you will have to go out later at night (8-10pm, for example). Put on comfortable shoes and go out to walk around your house for at least 1h. You can take your notebook or Ipad if this helps you take notes and remember better. When you come back, use at least another hour to describe the objects (in the wide sense of the term) with which your body has come in contact with during the walk. You can write in complete sentences or shortened phrases, paragraphs, lists. The important thing is to try to share the experience of the evening walk. Write at least three pages. Take at least one more day in revising the writing as many times as it is necessary. Use the spell checker. When you are ready, send your writing to en PDF format to your classmates by email.

EE#2: Getting lost in an unfamiliar neighborhood in Houston

For this exercise, you will need to choose a neighborhood in your city that is unfamiliar to you. Prepare for a 1h walk. Take your notebook or IPad and the neuroscience recording equipment. Try not to not make it noticeable. Let yourself be guided by the urban landscape. Maybe you can create rules: turn left when you see a red color, for example; or change walkways when you find high cracks on the sidewalk. The idea is to put attention to your surroundings, human and not human. From the architecture that defines that space to the people that appear, or not, in it. Do not try to obviate sensations of comfortability or likeness. Take not of the components of race, ethnicity, class, in this space. Listen to as many languages as are spoken there. Feel the temperatures that surround you. Take a map and annotate your path. Take pictures if that is convenient for your text. Gather objects or waste in your path. As you did before, when you get to your writing space, take all your written and remembered material. If you used maps, use them in your written exercise. If the collected objects are useful to you, use them in your text. Take not of the material produced by the neuroscience laboratory's recording equipment and, if you find that it can add to your piece, use it. Write at least 3 pages to share with the group. Send your final version before the weekend.

EE#3: Eco-writings

For this exercise you can move to the outskirts of the city or choose a place inside the city that keeps some natural aspects. As in other writing excercises, observe with care and annotate as much as you wish. You can register your experience while you walk and traverse the chosen area and / or when you take a break and come back to your usual working place. In either case, investigate your interior and exterior. Do not forget to write with all your senses. What is the relation of the city or the urban creation with the space you are currently exploring? Are the country and the city as different as we suppose? What type of creatures, human or not, habitate the explored space? What type of activities happen there or characterize that space?

Write up to a maximum of three pages in double space to share with the class.

EE#4: Walking together

In our societies it is increasingly less common to find opportunities to walk together with others; however, there are still some possibilities. Participate in a walk (it can be a protest walk) or a popular walk (it can be of religious character or not) and observe well your surroundings.

What are the causes for which the people walk in community? What is the background, the history and the expectations of this event? What is the feeling is perceived? What type of conversations are heard? If for any reason you cannot participate yourself in an event like this, identify someone that has participated in a special peregrination, interview that person, and write a narrative of the experienced based on that interview. Do not forget to add important information with respect to the history of this practice. Three pages, double space to share with the class.

EE#5

With the help of a map, draw the lines in your private city. Where do you move more? Every site has its own voice or voices, literally. The objective of this exercise is to listen as attentively as possible to what the people say in each of the nodes in which you stop with most frequency.

Design a series of stops and go there. Stop for a while. Listen with attention. In a notebook or in a screen of your choice, register what you hear without any discrimination towards order or origin. Use all the collected material to elaborate a piece as a lonely Walker.

EE#6

Recently, an african american swimmer won for the first time a gold medal in the Olympics.

Some reporters noted that the accomplishment was linked to the history of racial segregation in public swimming pools in the United States. In effect, a place apparently so innocent and dedicated to leisure can also respond to tense relations of power between races, classes, genders. To complete this exercise, you will need to visit a pool, preferably a public pool.

Observe with care. Go into the water. Make the annotations as soon as you can, even around the pool or in the dressing room. Is there a story to emerge there?

2 SUPPLEMENTARY DATA

The data pre-processing flowchart is shown in Fig. S1. The Generalized Partial Directed Coherence (gPDC) distributions for the Delta (Fig. S2), Theta (Fig. S3), Alpha (Fig. S4), Beta (Fig. S5), and the Gamma bands (Fig. S6) are displayed below. gPDC scores are displayed for each directed connection pair, across EEG recording sessions, marked as circles. Black: Preparation Phase. Blue: Generation Phase. A histogram and boxplot describing the distributions are shown in the lower panels. The dashed line indicates the 90th percentile of the PDC scores for the alpha band during the generation phase

2.1 Figures

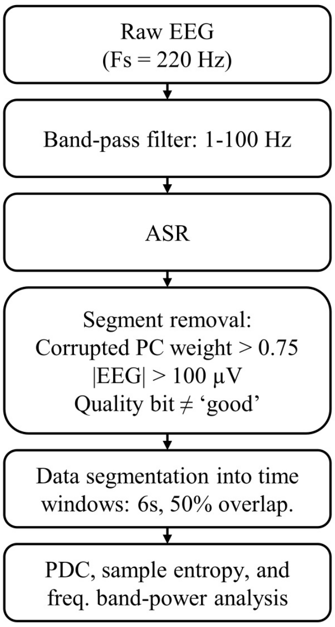

Figure S1. EEG data pre-processing and feature extraction. Flowchart for EEG data pre-processing and de-noising.

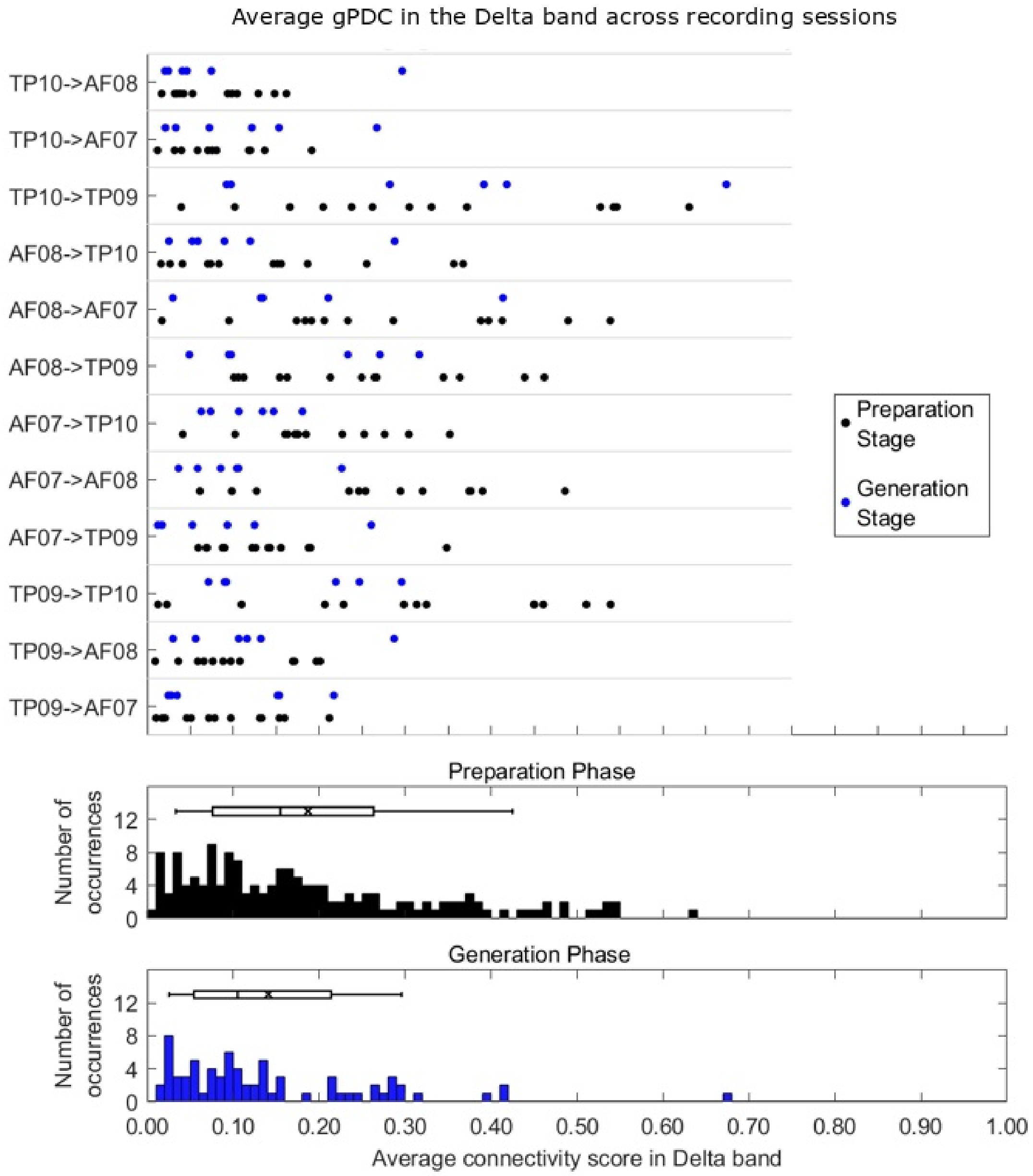

Figure S2. gPDC distributions in the Delta band (1-4 Hz). Average PDC for each directed connection pair. EEG recording sessions marked as circles. A histogram of the distributions is shown in the bottom panels.

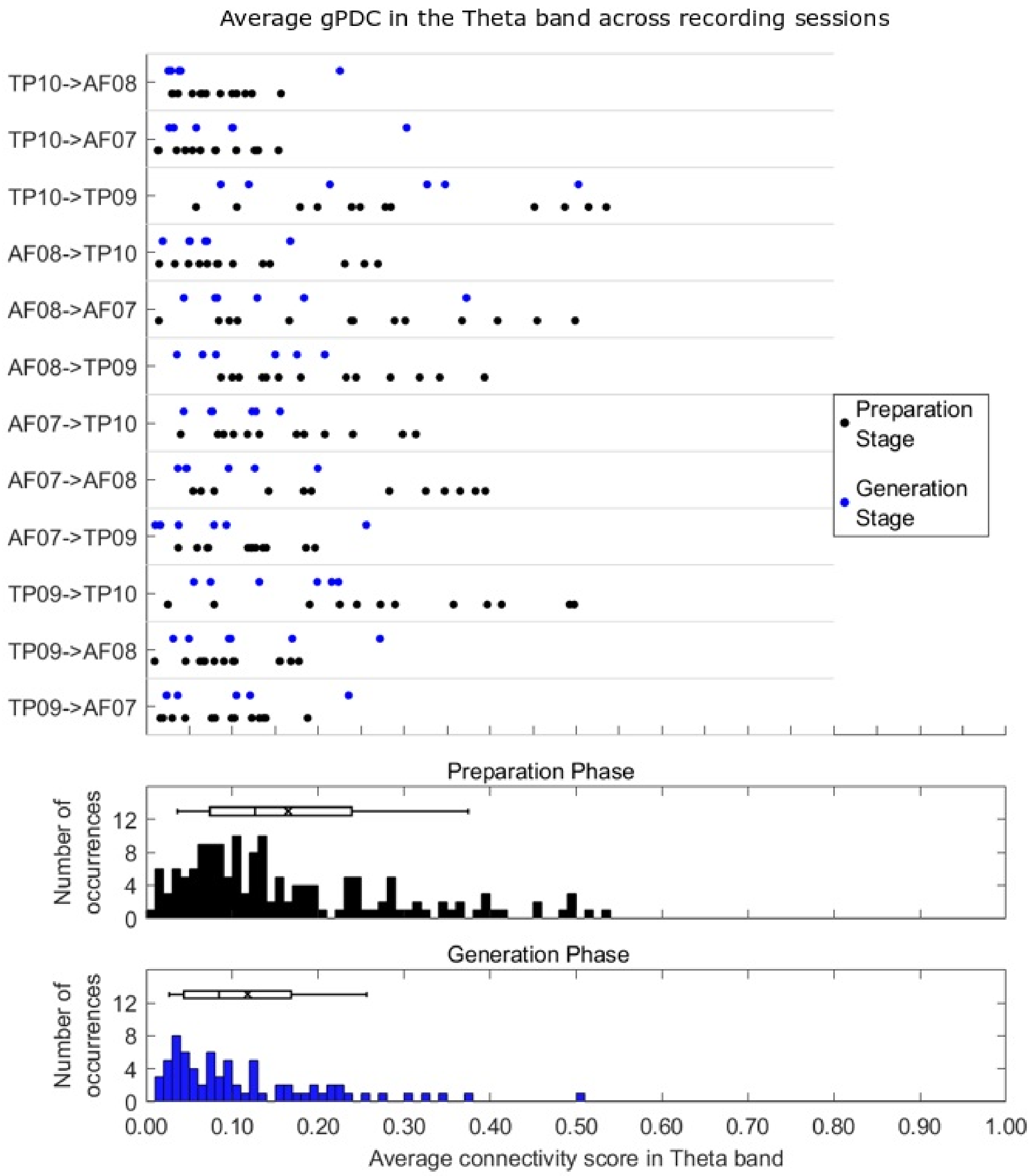

Figure S3. gPDC distributions in the Theta band (4=8 Hz). Average PDC for each directed connection pair. EEG recording sessions marked as circles. A histogram of the distributions is shown in the bottom panels.

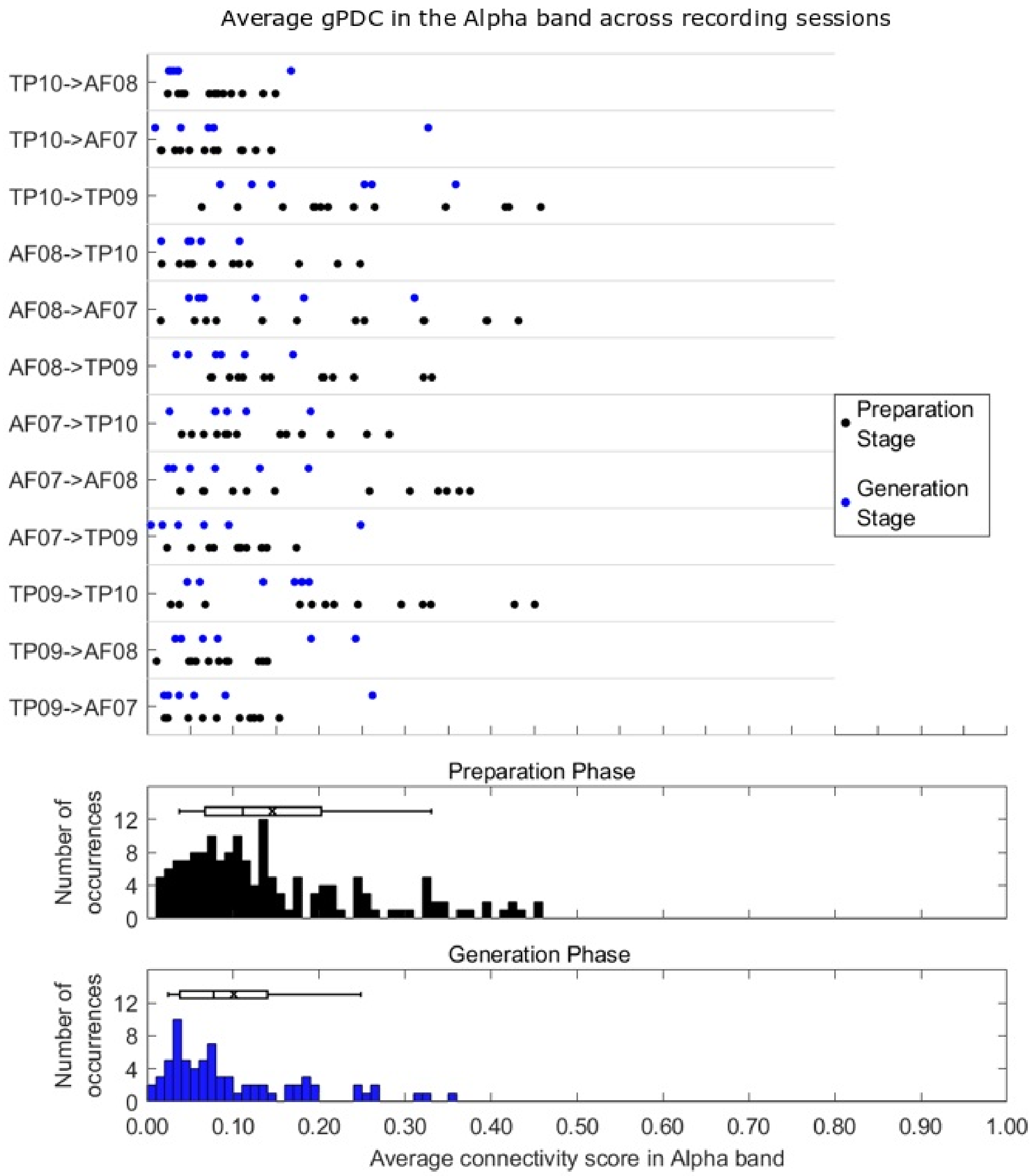

Figure S4. gPDC distributions in the Alpha band (8-12 Hz). Average PDC for each directed connection pair. EEG recording sessions marked as circles. A histogram of the distributions is shown in the bottom panels.

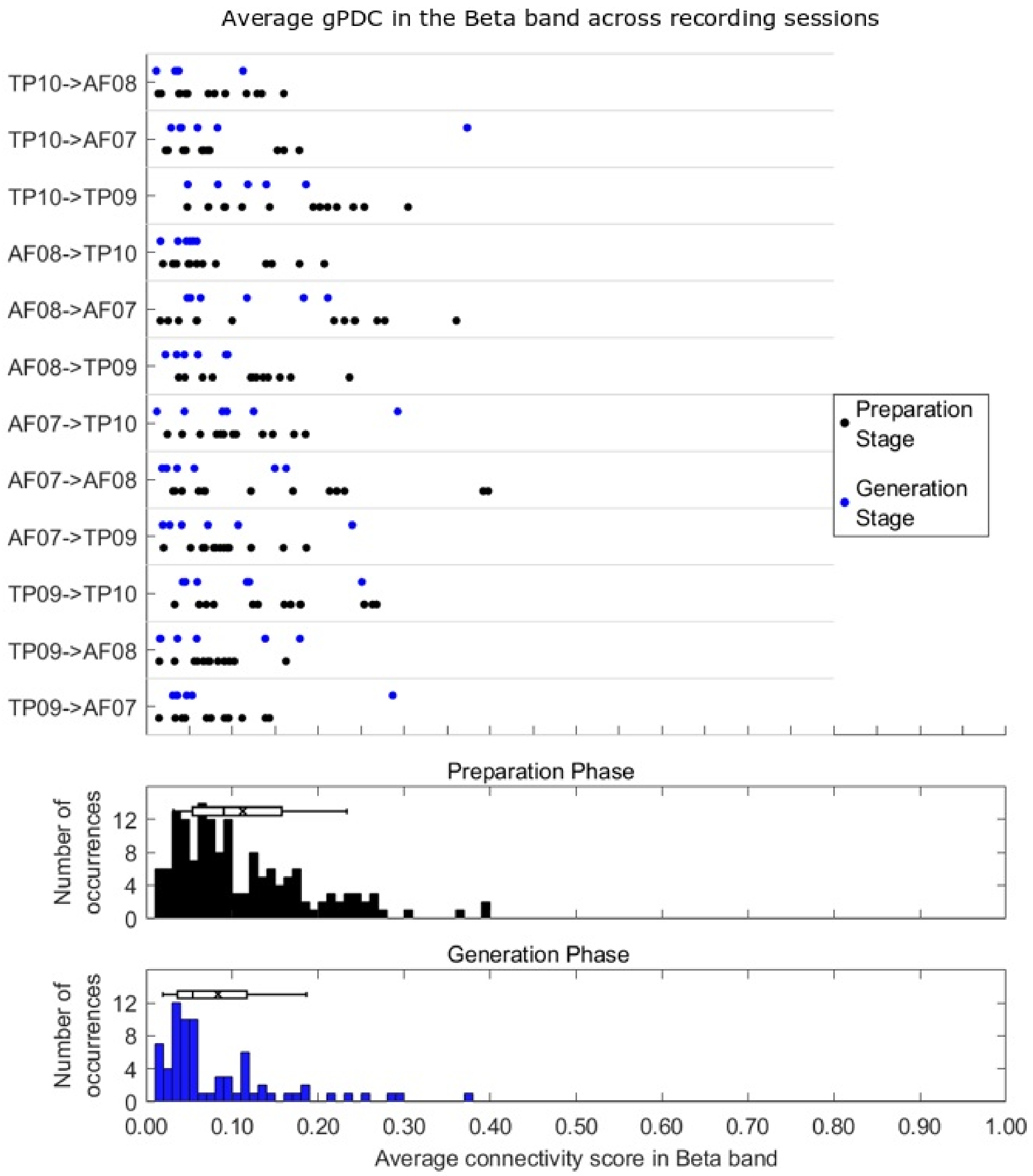

Figure S5. gPDC distributions in the Beta band (12-30 Hz). Average PDC for each directed connection pair. EEG recording sessions marked as circles. A histogram of the distributions is shown in the bottom panels.

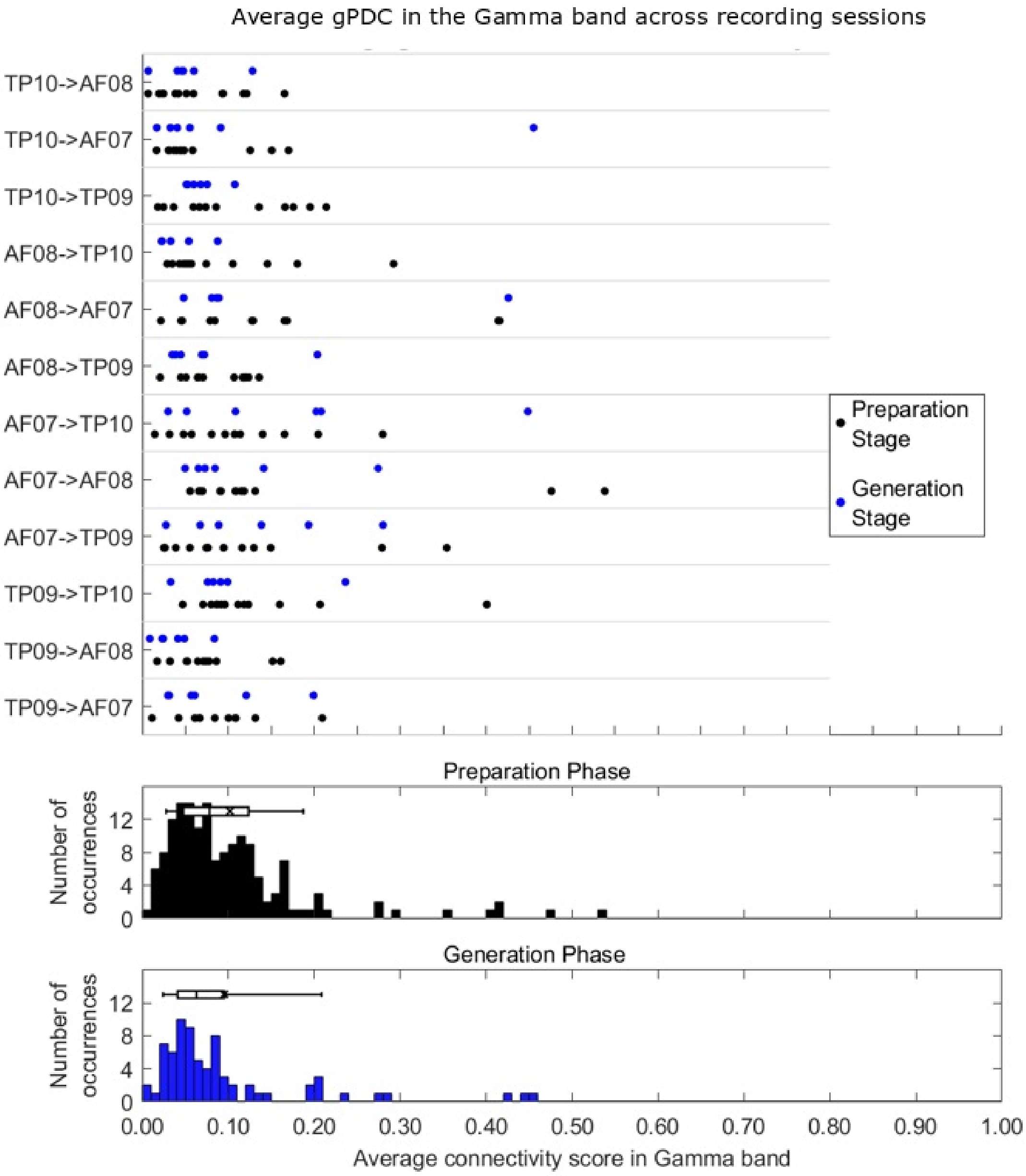

Figure S6. gPDC distributions in the Gamma band (30-50 hz). Average PDC for each directed connection pair. EEG recording sessions marked as circles. A histogram of the distributions is shown in the bottom panels.
